# Targeting Personal Recovery of People With Complex Mental Health Needs: The Development of a Psychosocial Intervention Through User-Centered Design

**DOI:** 10.3389/fpsyt.2021.635514

**Published:** 2021-04-08

**Authors:** Lisette van der Meer, Tessa Jonker, Heleen Wadman, Charlotte Wunderink, Jaap van Weeghel, Gerdina Hendrika Maria Pijnenborg, Ellie R. H. van Setten

**Affiliations:** ^1^Department of Rehabilitation, Lentis Center for Mental Health Care, Zuidlaren, Netherlands; ^2^Department of Clinical and Developmental Neuropsychology, University of Groningen, Groningen, Netherlands; ^3^BuurtzorgT Mental Health Institution, Groningen, Netherlands; ^4^Research and Innovation Center for Rehabilitation, Hanze University of Applied Sciences, Groningen, Netherlands; ^5^Department of TRANZO, Tilburg School of Social and Behavioral Sciences, Tilburg University, Tilburg, Netherlands; ^6^Parnassia Group, Parnassia Noord Holland, Castricum, Netherlands; ^7^Phrenos Center of Expertise on Severe Mental Illness, Utrecht, Netherlands; ^8^Department of Clinical Psychology and Experimental Psychopathology, University of Groningen, Groningen, Netherlands; ^9^Department of Psychotic Disorders, GGZ-Drenthe, Assen, Netherlands; ^10^Rob Giel Research Center, University Medical Center Groningen, University of Groningen, Groningen, Netherlands

**Keywords:** personal recovery, identity, sense of purpose, user-centered design, severe mental illness

## Abstract

Long-term admissions in psychiatric facilities often result in a gradual erosion of the identity of people diagnosed with severe mental illnesses (SMIs) into merely “patient.” Moreover, experiences of loss often reduced people's sense of purpose. Although regaining a multidimensional identity and a sense of purpose are essential for personal recovery, few interventions specifically address this, while at the same time take people's often considerable cognitive and communicative disabilities into consideration. This study describes the development process of a new intervention through user-centered design (UCD). UCD is an iterative process in which a product (in this case, an intervention) is developed in close cooperation with future users, such that the final product matches their needs. The design process included three phases: an analysis, design, and evaluation phase. In the analysis phase, the “problem” was defined, users' needs were identified, and design criteria were established. In the design phase, the collected information served as input to create a testable prototype using a process of design and redesign, in close collaboration with service users and other stakeholders. This resulted in an intervention entitled “This is Me” (TiM) in which service users, together with a self-chosen teammate, actively engage in new experiences on which they are prompted to reflect. Finally, in the evaluation phase, TiM was implemented and evaluated in a real-life setting. In a small feasibility pilot, we found indications that some people indeed demonstrated increased reflection on their identity during the intervention. Furthermore, TiM seemed to benefit the relationship between the service users and the mental health professionals with whom they underwent the experiences. The pilot also revealed some aspects of the (implementation of) TiM that can be improved. Overall, we conclude that UCD is a useful method for the development of a new psychosocial intervention. The method additionally increased our knowledge about necessary factors in targeting personal recovery for people with complex mental health needs. Moreover, we conclude that TiM is a promising tool for supporting people with SMI in redeveloping a multidimensional identity and a renewed sense of purpose.

## Introduction

People diagnosed with a severe mental illness (SMI; e.g., schizophrenia, bipolar disorder) frequently experienced meaningful losses [e.g., losing employment/housing/(romantic) relationships/dreams], because of their illness and lengthy admissions. Approximately 20% of people with SMI need these lengthy admissions in psychiatric hospitals or sheltered housing facilities (1–3), because of the severity and persistence of problems in multiple life domains [e.g., treatment-resistant symptoms (4), severe cognitive impairments (5), somatic health problems (6), poor self-care (7), and psychosocial dysfunction (8)]. Unfortunately, these lengthy admissions often lead to losing a sense of purpose, which makes formulating (and thus obtaining) long-term recovery goals extremely challenging (9). Despite the fundamental existential challenges this group of service users faces, the dominant focus during the lengthy psychiatric admissions is on symptom reduction and everyday functioning, so-called clinical recovery. Relatively little attention is devoted to personal recovery, which is the highly individual, often nonlinear, process of learning to live well, despite the consequences of a mental disorder (10, 11).

An important aspect of personal recovery is the transformation process of redefining one's identity from being illness-dominated to a multidimensional self that includes many other self-defining characteristics (12–15). Yanos et al. [(15), p. 2] defined this illness-dominated identity as “…the set of roles and attitudes that a person has developed about him or herself in relation to his or her understanding of mental illness.” The importance of (re)developing a multidimensional self-identity that goes beyond the illness identity is also highlighted in a conceptual framework for personal recovery in mental health, the CHIME framework, consisting of connectedness, hope and optimism about the future, identity, meaning in life, and empowerment (16, 17). Supporting service users in their process of personal recovery thus requires (among other highly related concepts as highlighted in the CHIME taxonomy) attention for the (re)development of a multidimensional self-identity.

Self-identity is considered to be an integrated construct and encompasses interpersonal and intergroup identities [Social Identity Approach; (18, 19)]. Interpersonal identity builds upon personal interests, attitudes, and behavior that differs from other individuals, whereas intergroup identity builds upon social group memberships (18, 19). Importantly, self-identity does not refer to one single intergroup/interpersonal identity; rather, it comprises a multitude of intergroup and interpersonal identities that are in constant internal dialogue and are highly embedded in a cultural and historical context (20, 21).

Several factors negatively affect self-identity in people in need of intensive and longer-term psychiatric services [see for a review (22)]. First, as a result of the difficulty to integrate the illness into a multidimensional self-identity, self-identity may be narrowed down to a more or less unidimensional self-identity of mental illness (14). This may be reinforced by the struggles of many people with SMI to self-reflect (23) and to express themselves because of cognitive impairments (24). Second, because of lengthy admissions, social isolation increasingly reduces self-identity to a unidimensional and illness-dominated construct (25, 26). Societal integration is challenging for people with SMI in general (27), and for those living in a residential care setting, the gap with society is even wider (28). Third, (self) stigma negatively affects self-identity if someone considers himself/herself part of a group that is devalued by society (18, 29). In their illness identity model, Yanos et al. (15) describe the impact a unidimensional and illness-dominated identity can have upon multiple recovery outcomes (e.g., hope, vocational outcomes, and even symptoms severity), particularly if combined with good clinical insight. In a recent review, Yanos et al. (30) demonstrated that in the past decade several articles have confirmed various components of their illness identity model, particularly the relationships between self-stigma and diminished self-esteem, hope, and impaired social relationships. However, this review also showed that studies specifically investigating (illness) identity are scarce, both in young adults in the early stages of mental illness, where identity development is still in full swing, and in service users in later stages of SMI. Because of the paucity of studies on identity development in SMI and because acquiring a sense of self is needed to be an active agent in one's own recovery process (31–33), both mental health research and practice should make the (re)development of a multidimensional self-identity a priority.

The literature indicates the availability of a variety of psychosocial interventions and tools that may help mental health workers to support service users with complex mental health needs in their recovery [see for an overview: (34)]. Integrated models of rehabilitation, such as the Boston Psychiatric Rehabilitation Approach [BPRA; (35–37)] and the Strengths model (38), provide tools to gain insight into and work toward recovery goals. Additionally, rehabilitation interventions that focus on recovery in specific (life)domains are available, ranging from cognitive remediation programs with a focus on functional impairment due to cognitive impairments [e.g., (39, 40)], lifestyle interventions [e.g., (41, 42)], and interventions aiming for recovery in the domain of work/school such as supported employment [e.g., (43)] or supported education programs [e.g., (44)]. In the last decade, user-led/user-developed interventions have gained increasing ground, such as Wellness Recovery and Action Planning [WRAP; (45)], “Recovery is up to you” (46), and Toward Recovery, Empowerment and Experiential Expertise (47). Additionally, digital technological developments, such as virtual reality, provide increasing possibilities for application in psychiatric rehabilitation interventions [e.g., (48, 49)].

Although these psychosocial interventions can be very useful in supporting the recovery process of people with complex mental health needs, these interventions do not explicitly address the recovery of self-identity from a unidimensional “patient only” construct into a multidimensional construct encompassing a wider range of self-identity defining characteristics. One psychological therapy that does incorporate the transformation of self-identity is narrative enhancement and cognitive therapy (NECT) (50). NECT is based on the principles of cognitive behavioral therapy and provides a structured group-based treatment using psychoeducation, cognitive restructuring, and narrative enhancement to target a reduction of internalized stigma that may result in an illness-dominated self-identity. The first results of NECT suggest a reduction of people's self-stigma in people with SMI receiving community care (51, 52) and people with schizophrenia-spectrum disorders receiving community care or partial hospitalization (53), although not all studies confirm the effectiveness of NECT in people with SMI in community or partial hospitalization programs (54). However, NECT still considerably calls upon cognitive and communicative resources of service users and may therefore be less feasible in people with SMI in need of long-term intensive psychiatric care. Another integrative psychotherapy that specifically focuses on improving metacognitive processing (which includes self-reflection) and the integration of a sense of self and others is metacognitive and insight therapy [MERIT; (55)]. Although the first results of MERIT are promising in terms of their effect upon recovery in people with schizophrenia and schizoaffective disorder in community treatment (56, 57), the therapy is highly verbal in nature, making it less feasible for the current target group.

In the present article, we describe the development of a new psychosocial intervention that aims to stimulate the (re)development of a multidimensional self-identity and that is closely tailored to the recovery-related needs of the service users with SMI. In order to do so, the intervention should follow the principles of recovery-oriented care, implying that it should be person-centered and strength-based [e.g., (38, 58)]. Moreover, the intervention should incorporate nonverbal components to meet the cognitive and communicative skills of the designated service users given their considerable impairments in these domains [e.g., (5, 8, 59)]. Examples of existing nonverbal methods include the use of photographs [Photovoice; e.g., (60)] or colored building blocks [e.g., (61)] to visualize a person's lived experience on a certain topic. Moreover, to optimize usability of the intervention, the needs of both service users as well as other stakeholders who support the recovery process (e.g., relatives and mental health professionals) with regard to the usability will be taken into account, as well throughout the development process.

In the development process, we adopted the innovative approach of user-centered design (UCD). UCD is an iterative design process in which the users' needs are central in each phase of the design process (62). UCD includes a variety of research and design techniques, such as the identification of the users and their needs, rapid prototyping, and design simplification (63–65). Although the advantages of the UCD process in the development of psychosocial interventions are increasingly recognized in digital therapies and interventions [e.g., (66–68)], to our knowledge it is only minimally applied in nondigital interventions or therapies. Nevertheless, the advantages of the UCD process are highly relevant for the latter type of treatment as the design goals, such as high learnability, efficiency, memorability, usability, and satisfaction, are very important for psychosocial interventions (64, 69).

It is part of UCD to explore the natural constraints to improve the implementability of an intervention. Therefore, applying UCD to the development of new psychosocial interventions may help to bridge the gap between research and clinical practice (64). This gap may arise from differences between the context in which the intervention is designed (e.g., a university) and the setting in which the intervention will be implemented [e.g., mental healthcare; (64)]. However, when such interventions prove to be efficacious, problems embedding the protocol in service systems, local circumstances, or other unforeseen complications (70) may limit effectiveness in routine clinical practice (71–73). The importance of considering these fundamental factors in the design of an intervention in the treatment of people with SMI becomes apparent upon looking at the success rate of implementing evidence-based treatments (EBTs) in people with SMI. Only 8–32% of the people receive EBT (74), and as few as 0–7% of the service teams offer EBT to more than 70% of the people serviced by the team, despite the availability of EBTs (75).

When looking at factors that impact this implementation success, the usability of the intervention seems to be of great importance [e.g., (64, 76)]. From the review conducted by Lyon and Koerner (64), particularly the design factors flexibility and complexity seem to affect the usability (and implementability) of psychosocial interventions. Flexibility refers to the extent to which an intervention can be flexibly used by (mental) health workers to accommodate individual service users. In the tradeoff between fidelity and flexibility, Cohen et al. (77) demonstrated that more flexible interventions seem to be better implementable, whereas interventions for which fidelity requires strict adherence without room for flexibly adapting to the context may be more difficult to implement. When interventions are too complex, they are also difficult to implement. Considering complexity is particularly important in the context of people with cognitive and communicative impairments because they should be able to understand and use the intervention and its components (78, 79). Proctor and colleagues (80) even suggest that factors such as the acceptability of interventions (e.g., costs, low complexity) may be equally important for successful implementation as treatment effectiveness. That is, interventions with lower effectiveness, which are more acceptable to stakeholders and are less costly, may ultimately achieve more behavior change than interventions with higher demonstrated effectiveness that are more complex and expensive.

The current article aims to describe the UCD development process of an intervention that targets the (re)development of a multidimensional self-identity in service users with an SMI and complex mental health needs, who need long-term intensive psychiatric care (e.g., in residential facilities or institutions for sheltered living). In addition, we will present the results of a qualitative pilot study in which we evaluate whether the developed intervention indeed targets the concept of (re)development of a multidimensional self-identity. Moreover, we will evaluate the feasibility of the intervention as well as determine factors that improve or impede its implementation.

## Materials and Methods

### Development of the Intervention: A User-Centered Design Process

**Figure 1:**
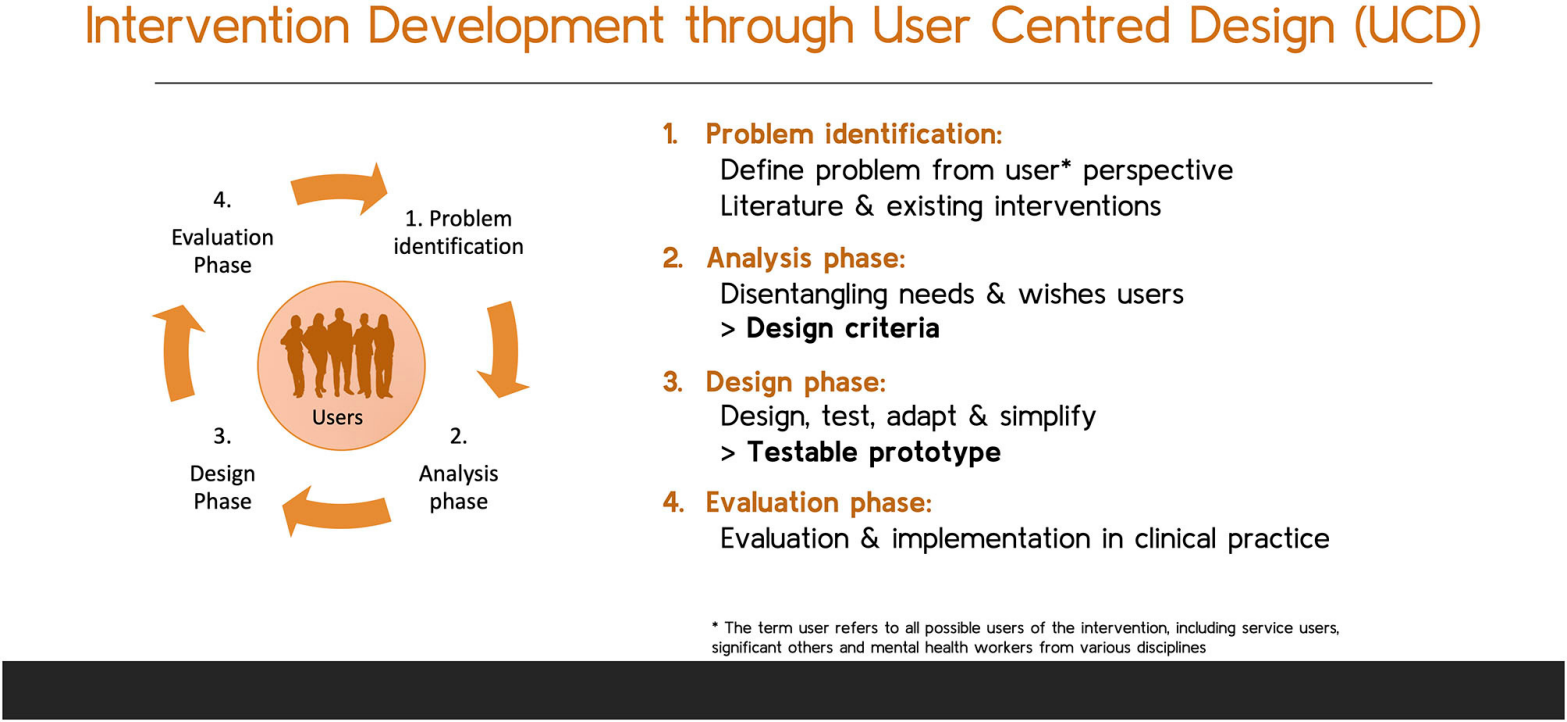
Visual UCD.

The first step in the UCD process was the formation of a design team, including a peer support worker, family peer support worker, and mental health nurse, alongside a junior and senior scientist, to ensure firm embedding of experiential knowledge in the process of intervention development. The design team was based on the grounds of a long-term psychiatric facility in the North of the Netherlands. In addition to the establishment of a diverse research design team, an ongoing process of expert consultation took place in the project. Upon formation of the design team, the UCD process commenced. Although UCD is a circular and iterative process, three main phases can be distinguished in the design process: (1) analysis phase, in which the primary components of the intervention were identified in close collaboration with the users; (2) design phase, which includes (re)design; and (3) evaluation phase, including implementation and evaluation in a real-life setting (see Figure 1 for a visual representation of the UCD cycle).

### The Analysis Phase

The analysis phase can also be regarded as the “empathy” phase, which is an important step in gaining a deeper understanding of users and their experience. Input for the design phase on specifically the topics identity and personality, life cycle, personal narrative, and wishes and goals was collected through individual meetings with service users, mental health workers from various disciplines, peer support workers, rehabilitation professionals, and professionals in the field of intellectual disabilities, as some of the cognitive and communication needs of people with SMI overlap with the needs of this group. Furthermore, we organized two focus group meetings [one meeting with service users (*n* = 5), researchers (*n* = 2), and one mental health nurse; one meeting with significant others (e.g., family members/friends; *n* = 2), mental health workers (*n* = 3), rehabilitation professionals (*n* = 2), and researchers (*n* = 2)]. All consulted informants emphasized the importance of the focus of the proposed intervention. In addition, they agreed that the intervention should be strengths based. The service user focus group demonstrated that the discussion in itself of topics such as identity was helpful for some participants. For example, when asked to describe her identity, one participant answered, “I do not have an identity, I sleep and that's about it,” whereas another participant replied by naming three positive traits that he noticed about her. This triggered her in realizing that she had a nonillness identity, which she forgot. This example also demonstrated that identity is a social construct, indicating that the process should include shared experiences (e.g., with other service users, family, friends, or healthcare professionals). Importantly, some service users preferred not to discuss their childhood or past, because of painful memories and trauma, whereas others like it as this provides others with information about “you as a person (who are your parents, where are you from, what do you like, etc.).” This indicates that choosing what (not) to discuss is a delicate matter and should be an individual choice.

The focus group with significant others (e.g., family members or friends), mental health workers, and rehabilitation professionals/researchers agreed to integrate the five life domains (work and education, social contacts, living, leisure, and finance) of the BPRA as a framework for the intervention [as proposed by the rehabilitation professionals; see for a review: (81)]. Participants agreed to add “health” as a sixth domain and to integrate “finance” in the “leisure and work” areas as financial aspects are often a means for further development instead of a goal in itself. Furthermore, the second focus group, as well as interviewed professionals in the field of intellectual disabilities, emphasized the importance of learning through experience, as discovering talents, qualities, and (dis)likes is often a result of undertaking activities. All participants agreed that experiences should not only entail past experiences and memories, but also include gaining new experiences. Finally, the second focus group underlined the importance of learning through using objects, in addition to learning through experience and language. Externalizing thoughts by using objects or visual aids can be particularly beneficial for service users with communicative and cognitive challenges [e.g., (60, 61, 82, 83)].

Summarizing the collected information from the various sources of information led to the following design criteria: (1) the importance of simplicity as the intervention should be suitable for use by service users, significant others, and healthcare professionals; (2) making use of a group process; (3) a strength-based focus; (4) focus on five life domains; (5) learning through using experience, objects, and language; and (6) equality of the service users and others supporting service users in their recovery process.

### The Design Phase

#### Brain Storm Session

A 1-day creative brainstorming session with two peer support representatives, one service user, one family representative, one music therapist, one art therapist, one psychomotor therapist, one psychologist specialized in the care for people with intellectual disabilities, one psychiatric rehabilitation professional, one student from the institute for positive technical design, two philosophers/artists, one graphical designer/artist, and three researchers was the first step in the design phase. The day followed the principles of “Design Thinking” in which several practical solutions are invented to solve a “problem” [see for a discussion how these principles can be applied to healthcare management and innovation: (65)]. Participants of the brainstorm session were challenged to think “out of the box” and to use their ability and experience regarding nonverbal means such as photography, art, or music and to find a solution for the main “problem”: “how can we help service users re-discovering their identities while meeting their cognitive and communicative needs?” The ultimate goal of this day was do develop a first prototype of the intervention based on the six design criteria established in the analysis phase. Participants proposed to frame the intervention as a “journey of discovery of my life” in which the service user chooses their own fellow “traveler(s)” (e.g., healthcare professional, peer support worker, relative, friend) and to use language to which people can relate instead of professional jargon. The nonverbal aspect of the journey consisted of collecting tangible and visual souvenirs along the way such as pictures or objects or using nonverbal tools such as pictures, foods, and smells to prompt memories.

#### Focus Groups Facilitating/Hampering Factors for Recovery

At this point, we aimed to gain important additional information from different perspectives regarding facilitating and hampering factors for personal recovery in an additional round of focus groups (three focus groups with peer support workers in training, who represented the service user perspective, two with mental health nurses, and one with family members). In this phase, we approached peer support workers in training, because of their trained ability to reflect upon their recovery process. These focus groups largely elicited similar information as the focus groups and interviews in the analysis phase, but revealed two additional design criteria that were deemed crucial by all three focus groups. First, there should be room for uniqueness of the service users' recovery process, and second, (self) stigma is an important barrier to the recovery process that should be addressed. Together, this culminated into eight important design criteria that should be considered in the development of the intervention.

The intervention should

1. be simple and intuitive;
2. allow for the use of a group process;
3. have a strength-based focus;
4. apply a framework of the life domains work, social contacts, living, leisure, and health;
5. facilitate learning through experience, objects, and language;
6. stimulate equality;
7. account for the uniqueness of each individual; and
8. incorporate the topic of (self) stigma;

#### Development of the First Testable Prototype

Based on the eight defined design criteria and during the brainstorming session developed design concept entitled “the journey of discovery of my life,” we developed a first testable prototype of the intervention, which we named “This is Me” (TiM).

TiM commences with the formation of a TiM pair, consisting of the service user and a person of their choosing (e.g., a relative, friend, mental health worker). In the following description of the intervention, we refer to the design criteria defined in *Focus Groups Facilitating/Hampering Factors for Recovery*. Together they undertake an activity aimed at reliving memories from the past or gaining a new experience (criterion 5: facilitate learning through experience, objects, and language). Possible activities are offered by the intervention along the five life domains (criterion 4: domains work and education, social contacts, living, leisure, and health). The content of the activities was designed in cocreation with service users, family, and mental health worker representatives and have a strength-based focus (criterion 3: strength-based). Examples of activities are “Visit your place of residence of in the past,” “Introduce the other person to your favorite music,” or “Teach the other person something you are good at.” In case the activity in question turns out to be unsuitable for (either one of) the pair, or for the moment, the pair will be offered a new activity. This way TiM remains attuned to the wishes of the individual participants (criterion 7: uniqueness of the individual). In addition, both participants have equal control because the activity is assigned based on chance, and neither participant is in the lead (criterion 6: equality). In accordance with the criterion of equality, it is also important that the activity is performed by both team members and not just by the service user (e.g., showing each other your favorite painting). The activities also include topics with the underlying theme (self) stigma and restoring old or establishing new contacts [criterion 8: (self) stigma]. The activities are formulated in a simple and action-oriented manner (criterion 1: simple & intuitive), so that the pairs actually enter into the experience (non-verbal) and not just engage in conversation (criterion 5; facilitate learning through experience, objects, and language). Finally, the journey should be captured and reflected upon by taking a picture or choosing an object that helps remember the specific activity. Although TiM aims to couple two people who engage in activities together, it is possible to organize the selection and sharing of experiences in a group process (criterion 2: group process).

#### User Evaluation Meeting

In an evaluation meeting with future possible users [service users (*n* = 7), a significant other (*n* = 1), peer support workers (*n* = 3), other mental health workers (*n* = 3), rehabilitation professionals (*n* = 2), and researchers/university teachers (*n* = 3)], we tested and evaluated this first prototype. Participants formed pairs, picked a card describing an unknown activity (switching activities was possible), were given 60 min to execute the activity, and were asked to reflect upon their activity. Participants indicated the activities positively influenced the equality between participants. Undertaking experiences aided the conversation between participants, even when participants knew each other. The reflecting questions were deemed unnecessary, as most of the topics were already discussed during the activity. Some participants indicated they felt uncomfortable leaving the institutional grounds, mostly out of habit or anxiety. Therefore, some activities may not be suitable for all users. Thus, the design should be adapted such that there is variation in challenges related to leaving the current destination and involving other people. Finally, participants felt that uniqueness was accounted for by the opportunity to choose pairs and the possibility to switch activities when desired.

#### Further Testing in Other Clinical Settings

**Figure 2:**
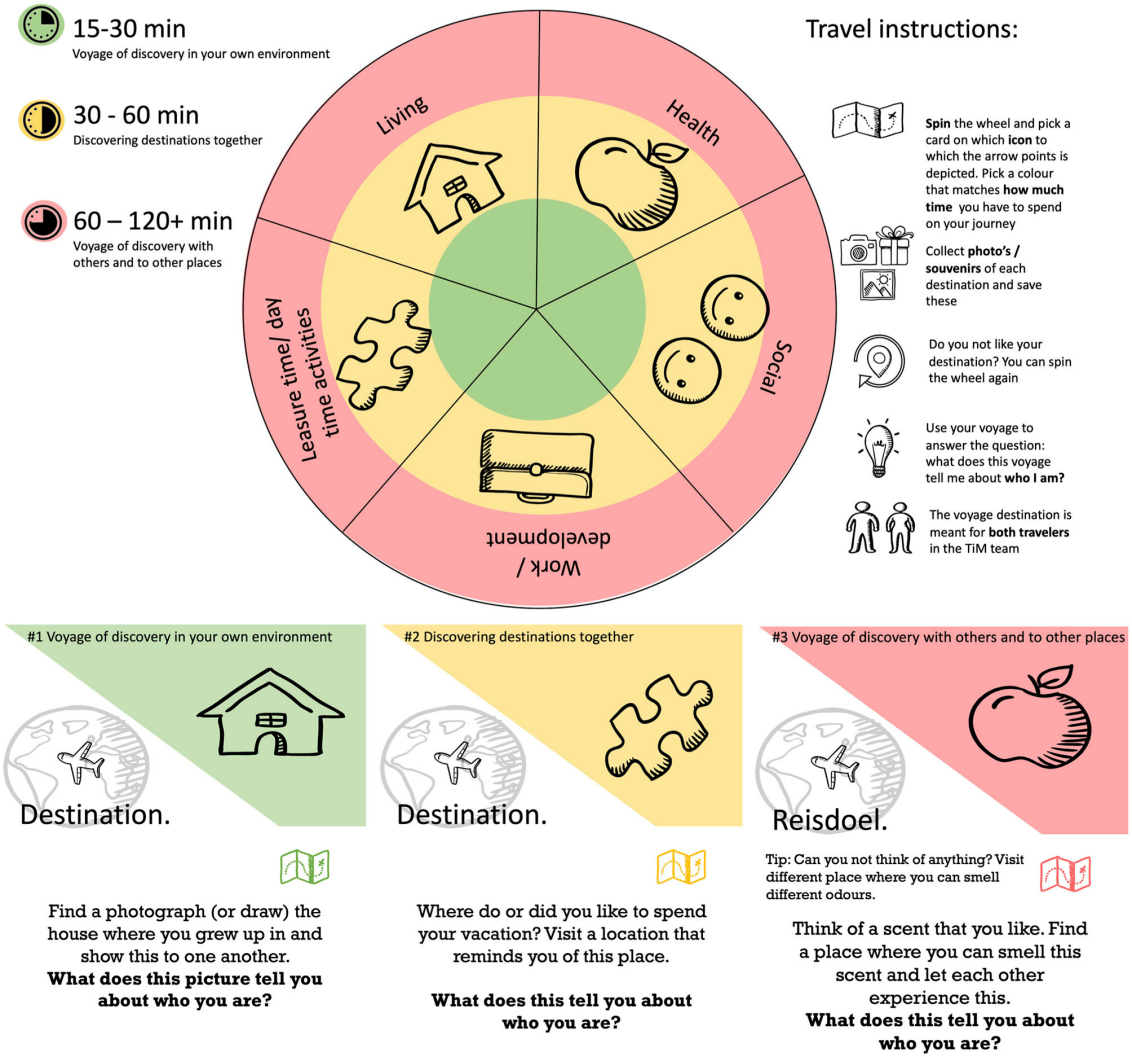
Second prototype.

Experiences of the user evaluation meeting served as input for fine tuning the prototype into a second prototype. We created more cards and activities in order to gain more experience in a clinical setting, where TiM will most likely be used. Moreover, to stimulate equality between users, we transformed TiM into a picker wheel, such that the category of the activity would be determined by chance (Figure 2). The categories represented the aforementioned five life domains (work, social contacts, living, leisure, and health). In this phase, in addition to content and form of TiM, the implementation procedure was evaluated in (1) group vs. pairs testing and (2) introduction at a random moment during the day, during a previously organized activity, upon invitation, and in an individual manner to pairs (service user & mental health worker). Five different locations/departments at two Dutch psychiatric rehabilitation facilities (four at Lentis Zuidlaren, in the North of the Netherlands; one at Dijk en Duin, in the West of the Netherlands) participated in testing the prototype.

Importantly, this round of testing taught us that the equality that people experienced in the user evaluation meeting was not always experienced in a similar way in a clinical setting. In many cases, the existing division of roles between client and care provider implied that (nursing) professionals took the lead when choosing activities. Although the picker wheel ensured that category of the activity was now determined by chance, the activities within each category were printed upon separate cards. In practice, mostly the care providers took the initiative in selecting a card, rather than it being a shared decision. Additionally, it appeared necessary to explicitly add the possibility to reject an activity and spin the wheel again in case the activity does not suit either one of the team members. Furthermore, testing the prototype at clinical departments revealed that people were tempted to remain seated and talk about, instead of actively engaging in an activity. We encountered barriers when the activity required people to leave the location. This was sometimes due to service users feeling uncomfortable to leave the home, but also for lack of time of nursing staff to undertake activities elsewhere. Therefore, for each activity, three “challenge” levels in terms of location and involvement of others were created. In terms of location, the most challenging level requires people to leave the premises, whereas the least challenging allows people to stay in their own home. In terms of involvement of others, the most challenging level requires the involvement of other people than the TiM pair, whereas the least challenging does not require this.

The collected results of the evaluation in this phase indicate that, despite the lessons learned, participants were enthusiastic about TiM. They like engaging in the activities, and in all cases, participants learned something new about their TiM partner, even if they had known each other for a long time. Participants also appreciated the opportunity to choose their own activity.

#### Designing a Third Prototype

**Figure 3:**
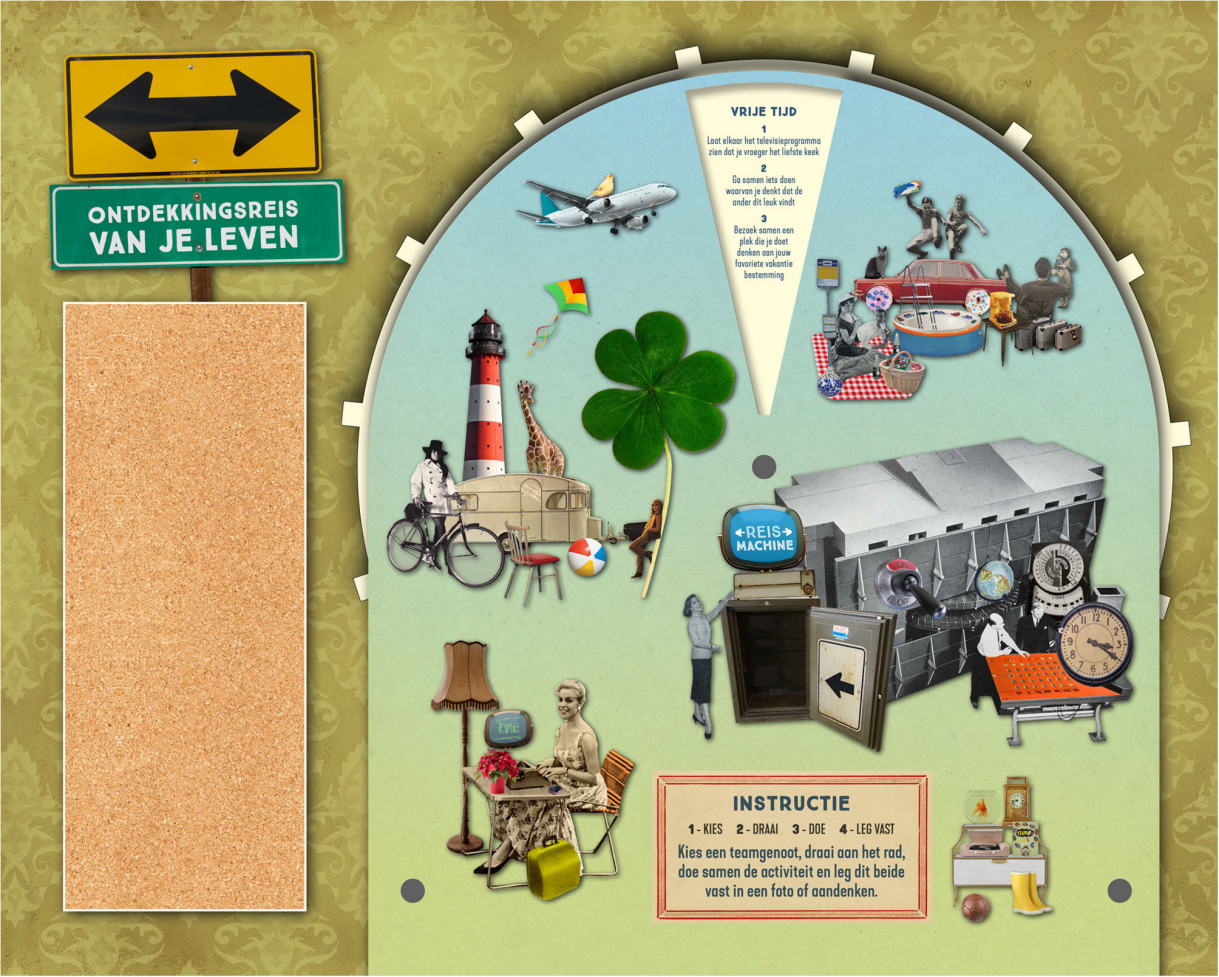
Third prototype.

We used the results from the previous rounds of testing to adapt TiM into a third prototype. To ensure an attractive and intuitive design, we appointed a graphic designer/artist (present during the brainstorm session) for the prototype design. We transformed the prototype into a larger picker wheel (Figure 3), now requiring a standing position and thus stimulating users to adopt an active posture. We also designed a travelogue that TiM team members could use to log (1) with whom they undertook the activity, (2) which activity they undertook, (3) how they experienced the activity, and (4) their experience through a souvenir (Supplementary Material 1 for an impression of the travelogue). The latter was included to commemorate the experience and reflect upon it by assigning thought and emotions to the experience, as well as the opportunity to share the experiences with others. Moreover, in this third prototype, the activities within the categories were no longer presented upon separate cards, but rather on the picker wheel to ensure the choice of activity would not be decided by the care provider.

Based on the UCD process up to this point, we integrated the “TiM training” in the design for two main reasons. First, simple instructions are imperative to guarantee accessibility for all possible participants (clients, relatives, significant others, mental health workers). In addition, a “professional training” may bring about an unfair advantage for professionals and create inequality between users. Therefore, the following simple instructions were printed on the wheel and in the travelogue: (1) Pick a TiM partner, (2) Spin the wheel, (3) Do the activity, and (4) Log the activity in the travelogue.

#### Evaluating the Third Prototype

##### Evaluation Meeting With Users of TiM

The third prototype was tested in two separate group meetings, organized at a central location in two long-term clinical facilities, such that service users could also decide to participate on the spot. Participants included service users (*n* = 9), mental health workers (*n* = 5), students' user-centered design (*n* = 4), a significant other (*n* = 2), a researcher (*n* = 1), peer support worker in training (*n* = 1), and the graphic designer responsible for the prototype. The responses indicated that the wheel was inviting and stimulated participants to spin it and read the activities. The appearance of the wheel also triggered the curiosity of nurses and service users who passed by, some of whom participated (*n* = 3). Nevertheless, a number of critical points did emerge. First, participants were confused by the word “travel destination” that we initially used instead of the word “activity,” which they associated with a vacation or actual journey. Second, the wheel simultaneously presented three activities, each representing one of five life domains and each at a different challenge level. This was confusing for participants; they preferred organization of activities per theme and to apply the three levels of challenge based upon this activity. Third, the different print color for each challenge level hampered the readability. Fourth, the instructions and game rules page in the travelogue caused confusion, inactivity, and a focus upon understanding TiM instead of engaging in activities. Finally, the verbal nature of the travelogue was not suitable for all participants. Participants preferred a visual log and use photographs, drawing, etc. Finally, two individual TiM pairs who tested TiM separately most importantly indicated that the various activities often resulted in meeting people who (have) play(ed) an important role in the lives of the service users.

##### Consultation of Rehabilitation Professionals

At this stage, different professionals in the field of research on recovery and rehabilitation (*n* = 6), a peer support worker (*n* = 1), a family peer support worker (*n* = 1), and social innovators (*n* = 2) were consulted to address final issues encountered with the third prototype. First, reflection upon experiences remains a difficult process for the service users as well as their TiM partners. The various professionals emphasized that verbal reflection may not be feasible, especially as most service users experience difficulties in verbal communication. They noted that not the learning experience, but rather the experience itself should be emphasized to stimulate personal recovery. Reflection upon the experience can be facilitated by enabling users to visually capture the experience (e.g., by taking a photograph) and by valuing this experience (visually). This value assignment should not be an emotional reflection but rather an appreciation of the moment (I liked this experience) that may include a short elaboration why they did (not) appreciate the experience.

Another difficulty we kept encountering was how to activate participants to go out and experience smells, music, interactions, or other activities. The professionals agreed that the activities/experiences should be actively formulated (“visit place X, listen to music, taste food”) to make TiM more action oriented. Furthermore, both service users and staff often have a fixed routine and behavior. From our own experience with TiM up to this point as well as from the experience of the professionals, we learned that “breaking routine” is a great way to activate participants and trigger behavior out of the usual pattern. For example, when we tested the prototype in a festival-like meeting outside a clinical setting, most participants were likely to join, to experience, and even to reflect upon the experience. However, when we used the prototype inside a clinical department, people showed mostly the behavior that confirmed the role of patient vs. healthcare professional. Through making TiM movable and placing it on the locations only temporarily, we prevent TiM becoming a fixed object in the usual setting. Thus, TiM can remain to “disrupt” the environment to some extent, attract attention, and possibly trigger different behavior. Finally, the professionals suggested to create different designs of TiM, for example, a pocket-size edition, to facilitate the use of TiM out of a clinical setting (e.g., use by relatives).

**Figure 4:**
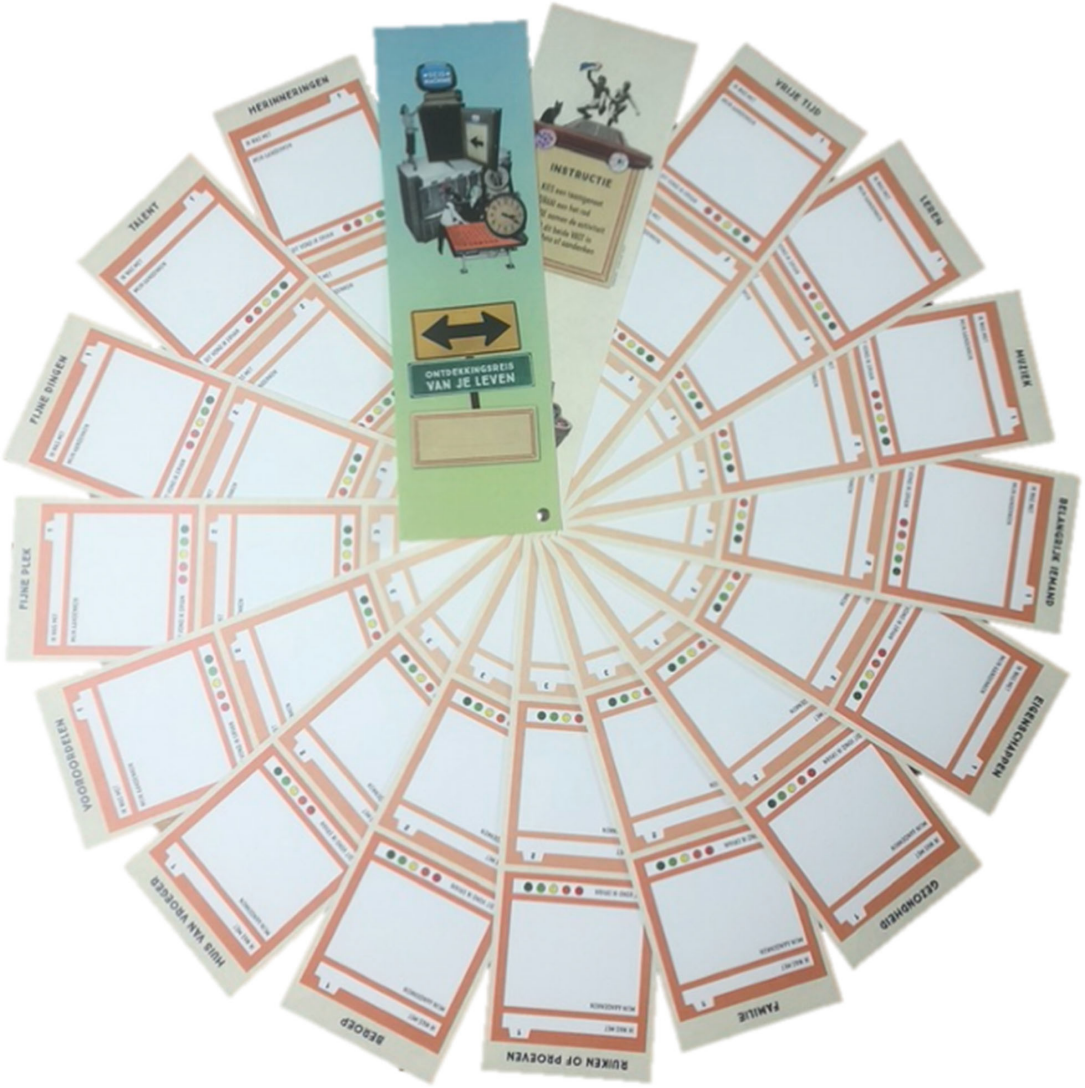
Hand fan travelogue.

Based on the second evaluation meeting and the consultation of rehabilitation professionals, the form and content of TiM were fine-tuned, and a pocket edition was made, which was essentially a smaller version of the large picker wheel. The Supplementary Material 1 provides a complete overview of activities that were included in the final version of the intervention. Additionally, the form of the travelogue was adapted and now resembled a large handheld fan. The travelogue was organized in the exact same way as the large picker wheel, such that when laid out completely, it formed a large circle (Figure 4). The travelogue allowed people to now visually represent the activity and value the experience.

### The Evaluation Phase: A Pilot Study

In this phase, we implemented and evaluated TiM in a real-life setting through organizing an implementation tour at 16 residential care facilities for people with SMI in the north of the Netherlands. This entailed a festival-like (including music, food, drinks) introduction event at each location to create an easy-going and activating atmosphere that stimulated other than usual role patterns and to maximize attendance of all parties. At the event TiM was introduced and explained. People had the opportunity to use TiM for the first time and to gain, share, and exchange their experiences. All potential users were invited to these events: service users, family and significant-others, and mental health professionals. At 3 of the 16 locations (one city and two rural), all service users who were present at the festival-like introduction event (*n* = 30) were additionally invited to participate in a qualitative evaluation study. They received verbal information about the study, and if they indicated to be interested, they also received a written information leaflet with additional information. After 1 week, a researcher revisited the location to answer questions. All service users were allowed to participate in TiM, regardless of their decision to participate in the pilot study. Before signing informed consent, participants were informed that they could withdraw from the study at any given moment. Service users received a gift certificate of 15 euros at the start of the study, and another one of 15 euros at the end of the study. The Medical Ethical Testing Committee at the University Medical Center Groningen provided ethical clearance for the study.

#### Participants

Eleven service users with an SMI (out of the 30 approached) who live in a sheltered living environment agreed to take part in the qualitative evaluation study. One service user withdrew from the study before the interview (reasons unspecified). Of the 10 participating service users, six were female and four were male; the median age was 52.5 years (range, 29–61 years). On average, service users had lived 14 years (range, 1–30 years) in sheltered living facilities or other clinical psychiatric settings. Self-reported diagnoses included schizophrenia, schizoaffective disorder, autism, bipolar disorder, borderline personality disorder, anxiety, and depression. In addition to the service users, three mental health professionals (one social worker, two peer support workers in training; age range, 30–38 years old), who used TiM with the service users, were interviewed. The mental health professionals also signed informed consent, and the service users gave written permission for the professionals to share information about them. Service users and mental health professionals were instructed to use TiM at least once every 2 weeks. However, we did not further control this, as we were interested in the natural usage of TiM.

#### Materials and Procedure

The semistructured interviews for service users and mental health professionals were created with four questions in mind: (1) how do people use TiM in practice; (2) what is the effect of TiM on the service user, more specifically on identity development and the relationship with the TiM partner; (3) how do service users and mental health professionals evaluate (the separate components of) TiM; and (4) which factors influence the implementation of TiM. The interviews were created by the research team in two consensus meetings. To evaluate to feasibility of the questions of interview, one service user who had used TiM, but did not participate in this study, was consulted for feedback. The questions were only used to guide the interview; participants were free to share their own experiences. Interviews were conducted face-to-face in a quiet room at the sheltered living facilities. The interviews were conducted by a master student in clinical neuropsychology, who also worked as a clinical intern in a residential setting for people with an SMI. In some cases, a mental health worker was present because the service users wanted their support as they did not know the interviewer. The interviews were recorded such that they could be transcribed.

#### Analysis

Thematic analysis in Atlas.ti 8.0 for Windows (Scientific Software Development GmbH) was used to analyze the transcripts. The analysis was carried out by two authors (LvdM and EvS) who represent different perspectives (respectively, a researcher in the field of SMIs and self-reflection, and a researcher with experience as a mental health service user). *A priori*, the main themes *Usage, Effects, Evaluation*, and *Implementation* were defined, as they relate to our research questions. *Usage* refers to the manner in which people used TiM, including which subcomponents people used and the usage frequency. *Effects* refers to changes people noticed as a result of their usage of TiM. We anticipated the subthemes *Identity* and *Relationships* within the main theme *Effects*, as identity was the target of TiM, and identity change occurs in a social context. *Evaluation* refers to the appraisal of (aspects of) TiM. *Implementation* refers to the circumstances influencing (successful) usage of TiM. Next, EvS did the first round of coding using the Noticing-Collecting-Thinking method described by Friese (84). A recursive strategy was used during this process. After grouping the codes into the main themes, EvS and LvdM discussed and interpreted the results. Finally, EvS did an additional round of (re)coding and (re)grouping, including the induction and division of subthemes, based on this discussion.

## Results

No new main themes were derived by induction, in addition to the *a priori*–defined themes *Usage, Effects, Evaluation*, and *Implementation*. Under the main theme *Effects, Activation* was found as a new subtheme, next to the subthemes *Identity* and *Relationships*. Results will be presented per theme, although it should be noted that themes were linked (e.g., positive effects were reasons for positive evaluations; better implementation was associated with more usage, etc.); therefore, links between themes will be described as well. All quotes were translated from Dutch to English. For privacy reasons, proper names were removed, and the pronouns *she*/*her* will be used for third-person singular.

### Usage of TiM

Six of 10 service users participating in the pilot study used TiM after the introduction event. The frequency of usage varied from often (five or more times; *n* = 3), to regularly (3 or 4 times; *n* = 2) to once (*n* = 1). All service users who used TiM used it with a mental health professional. Two other service users formed a TiM pair together, but did not use TiM because the activity they had planned was no longer possible because of a physical disability of one of them. As they were not assisted in planning something else, they abstained from using TiM. The final two service users did not use TiM because of health problems and because they were not reminded by the staff to use TiM; the latter becomes clear from the following statement: “Nothing was said to me, nobody said anything. If that does not happen, I don't do it on my own accord, because I don't know how that thing (the TiM picker wheel) works.”

Some people also used TiM in a group in addition to individual meetings (*n* = 3), by taking turns in answering questions or doing an activity together (e.g., sharing their favorite music, visiting an outdoor workout park). In other cases, TiM was used one-to-one, and activities took place both inside and outside people's living environment (e.g., visiting the parental house or a relative, visiting a Buddhist monastery, a digital tour of someone's former place of residence, meditating together). In one case, family was involved; they made a short video of the service user's former place of residence, including the old house and school. The travelogue was not used at all locations. One participant said she “did not see the point” of documenting the activities in the travelogue. However, at another location, people used the travelogue instead of the wheel to select activities, because they used TiM frequently and spinning the wheel did not provide them with sufficient variation in activities.

Despite the intention of establishing equality, it was usually the mental health professional who took the lead in the usage of TiM. For example, when picking one activity out of three alternatives, one professional noted:

“It can be a little bit difficult to make a choice out of three things. I left that choice as much as possible with <name service user>, but sometimes you have to make that choice yourself because she found it somewhat difficult.”

At another location, a service user described that the mental health worker usually proposed an activity, but always consulted the users whether they liked the idea. Thus, while the professional took the lead, service users still had an active role in the choices. Spinning the picker wheel itself was done by both the service users and the mental health professionals.

The type of personal information that professionals were willing to share with service users varied. While one professional shared only “superficial things,” another shared a lot of personal information, especially related to childhood and family. When asked whether she found it difficult to share personal information, she replied:

“Not for me personally, I am also a peer support worker in training and then you are more inclined to use personal things in your work anyway. And I find it especially nice that there is more equality in that relationship, or something. I find it harder to navigate when there is more distance. I don't find it difficult, but I can imagine that it is different for other people.”

The amount of personal information service users shared with professionals also differed. This may be a personal preference according to one of the professionals: “then I come inside her head, and then I do too much with her… she has that with a lot of people.” The personal information shared by service users did not necessarily correspond with what professionals shared, as some service users still shared in-depth personal information when the professional did not.

### Effects of TiM

Within the theme *Effects*, we anticipated *a priori* the subthemes *Identity* and *Relationships*, and inductively we also found the subtheme *Activation*, which contains statements about becoming more active as a result of TiM. Regarding the subtheme *Identity*, one service user was very articulate about how TiM helped to deepen and broaden the way she saw herself: “In a certain sense it clarifies things about yourself, you can put things in order… That is nice to know, because maybe you come across thing you never knew about yourself.” Furthermore, TiM made this person reflect on her past: “I came more in touch with my youth, I used to think about that already, but now I dwell upon it more.” Other service users described how TiM made them think about their past, especially activities such as visiting or drawing their old house or talking about family. Some people said they were emotionally touched by these kinds of activities; someone stated: “You do come across yourself, across your own things; they come up, yes you do feel things….” The most memorable TiM activities that service users reported were activities where people were physically active, like visiting their old house, meditating together, or visiting an outdoor work-out park, although most people said they did not think a lot about the TiM activities. Four service users indicated that TiM did not lead to perceived changes in the way they saw themselves, although one of them said that she still learned something new about herself, namely, “That I should look at the positive aspects and to use my capabilities… That I do not diminish or sideline myself, those kind of things, I am allowed to be there.” Regarding the subtheme *Relationships*, four service users mentioned that relationship with the TiM partner became better, closer, and more equal. Another person indicated that the process of getting acquainted was much quicker: “That same connection between me and my mentor, that took more than 2 years, before I thought I can tell them about this and that. With <name TiM partner>, it was two/three weeks.” For this person, TiM worked very well to get acquainted with a new mental health professional. The fact is that TiM offered this service user and professional topics where they could talk about “worked wonders.” The mental health professionals also noticed changes in the relationship with the service users. One mental health worker described the contact as a result of TiM with the service user in the following way:

“Much more, well… not really like you have become friends or something, but much friendlier or something. Much more open, easier, barrier-free so to say. That you have made a little bit of a connection in that sense. Because you share things of and with each other.”

This mental health worker also noted a change in roles that resulted from the service user learning the professional mediation skills:

“Normally you are a bit in the role of learning or educating someone about something, or something, and that role turns around a bit. I have noticed about <name service user> that the contact has become much more equal, or something.”

This role change was also noticed by the service user who saw the professional differently: “I have seen the possibility that I can see <name TiM partner> as a common-meditator, or something what every that may be, because she also meditates.”

Regarding the third subtheme, *Activation*, a professional clearly described changes in the behavior of two service users with whom she used TiM. They would more easily approach her, take initiative, and ask questions. When discussing the openness of service users during a TiM activity this professional said:

“Oh well, that parental house, there she was very open and honest. Then she told me much more about her past than she normally would. And she said, how did she say that… nobody has ever done this with me. So, you do get appreciation. And they ask can we again sometimes…”

Two service users indicated that TiM had sparked their interest in certain activities. For example, a service user who previously visited an outdoor exercise park said that when cycling through that park, she thought: “Well, let's try that again sometime.”

### Evaluation of TiM

Most people indicated that it was “fun” to work with TiM. Most service users did not elaborate much, for example, someone said doing TiM was “a nice way of keeping yourself busy.” Someone else said she liked it because TiM offers some distraction, and she liked that she was working on the project with others. The service user who used TiM to get acquainted with a new care provider liked that there is no pressure behind TiM, the fact that TiM sets the topic for a conversation, and the element of chance. None of the service users evaluated TiM overall negatively. All service users were positive about the fact they had to use TiM together with someone else.

The mental health professionals were generally positive about TiM as well, one professional said:

“I am very enthusiastic about the project myself and then you pull residents into it. If you find something very nice yourself it will spread to the residents, because now they already come with things themselves.”

Another professional praised the visual aspect of TiM:

“Well, you make things visual, that is actually always good. Because they are actually very abstract concepts, an old hobby that you have lost, quite abstract, but with this you can make it visual very well. Uhm… I only see the benefit of it.”

A number of components of TiM received a mixed evaluation. Some people really liked the travelogue and used it to select activities, whereas others found it redundant. People generally liked spinning the picker wheel, but some service users found it difficult to pick one (of three) activity the wheel provides. Furthermore, one service user indicated that improving some activities on the wheel should be rephrased, and one professional stated that there was too much similarity between activities.

The first impression of TiM yielded anxiety in some service users, as they initially expected that it would be difficult. Someone who ended up not using TiM said: “If I look at it, it looks really complicated.” However, someone else ending up not using TiM, but who still intended to do so, indicated she would like TiM because “You do things that you otherwise would not do, not so easily. It's playful so there's no pressure behind it, so that's okay.” All professionals and all except one service user found TiM easy to use once using it. A service user summarized: “The wheel itself was wonderfully simple. 1, 2, or 3. Okay if we've already had this one, then we'll continue with two.” The person who still experienced difficulties using TiM had particular problems describing the activities she undertook and did not comprehend all the text on the wheel. Most service users said they would recommend TiM to others. When asked why, the service user who had been most articulate about the effect of TiM on identity said, “Well, it forces you to think about yourself, and then you might come across new things and that is quite nice.” Two other service users were more ambiguous; they believed everyone should personally decide whether or not they wanted to use TiM.

### Implementation of TiM

Within the theme of *Implementation*, several factors were perceived as barriers to or facilitators for the usage and success of TiM, some of which have already been described. First, with regard to barriers at the service user level, TiM was not used when (mental) health reasons or other problems were too prominent or urgent in a persons' life. Furthermore, not knowing how TiM works and a first impression that TiM is too complicated may be barriers to using TiM. Some service users had difficulty selecting an activity from the three options or to come up with an alternative activity when the originally planned activity was no longer possible. Finally, a service user said that the barrier to ask someone to do TiM was too high for her, especially as she was already otherwise engaged.

Some personal characteristics of the service users may also influence the effect of TiM; people may not like it when people come too close, have difficulty to recall experiences due to memory impairments, have difficulty reflecting upon their experiences with TiM, or are hesitant to reflect: “I found it quite nice, but it also made me a bit hesitant, because you are going to think a lot about yourself and I find that quite difficult.” Thus, as one of the service users remarked, TiM may not be for everybody: “You have to agree with it, it has to be something for you. You should think about it well before you start.”

Another important barrier in using TiM was the lack of someone to use TiM with, as staff was not always available or willing to participate. One service user said that using TiM in a group was not optimal for her: “Because there are more who get a turn, because you have to consider others, then you only have a small opportunity to tell something about yourself.” Sometimes, activities were difficult to organize and require substantial planning, a point raised by both a professional and a service user. This may lead to the abandonment of plans or require flexibility, as one professional phrased this:

“If they really want something… like going to the parental house, then it takes me a few hours… then I need to come back for it, and it costs a few hours of my time… but I write down those hours, it is not really a bother.”

Professionals found it difficult to be the only one in the team who used TiM, as nurses and carers were already otherwise engaged. Although according to one professional, this may also be a matter of perspective:

“For them it is more the idea that it is something extra, that it is not really required and then there is no time for it. While it really can be a nice component of your work, and it is not really seen in that way.”

The first facilitator indicated by one service user was the introduction event: “that indeed made the next step with <name Tim partner> a bit easier.” Regarding how service users should be approached to engage in TiM, one service user said: “Just approach people really carefully, because they often have a certain image, something like what has someone else got to do with what I know. So, first convince and then the person has to decide for themselves.” Another service user said that using TiM should be an individual's choice and not be obligatory. Service users who used TiM as well as those who did not said regular appointments would improve the usage of TiM, because “If I have to do it on my own accord, it will not happen, I know that about myself.” This corresponds to the finding that generally someone other than the service user needs to take the initiative, a point raised by multiple service users and professionals. According to a service user, the person you use TiM with should not be just anybody:

“Well, it is good to do it together with someone, and then with somebody who is interested in you doing it. Because if it is somebody who just sits there, then they are of no use. You have to do it with somebody who thinks along.”

Both peer support workers in training who were interviewed suggested TiM would be especially appropriate for use by peer support workers, given their different relationship with the service user. They all did not agree that TiM would be appropriate to use by peer support worker in training. For one person, TiM was part of her learning goals, giving her more room to use TiM, whereas it was difficult for the other to use TiM alongside the large number of assignments within her internship. One peer support worker in training indicated that other colleagues should also make time for TiM, because “Such conversations can bring you a lot in the long run and can also bring the clients further, I think.” Finally, a professional noted that the importance of positioning TiM within a team of professionals:

“So maybe it is an idea that these methods, TiM, are less profiled like a standard method, but more like an extra tool that can be used, and then it must continue to receive attention.”

## Discussion

The aim of the current article was to describe the UCD process and the qualitative pilot study of an innovative psychosocial intervention to support recovery of a multidimensional self-identity in people with an SMI. Most importantly, the process of UCD proved to be a thorough and inspiring process that leaves ample room for adaptation and improvement according to wishes and needs of its users (be it service users as well as people supporting service users). The pilot study demonstrated that the final product, TiM, seems useful and promising. Service users indicated that they enjoyed TiM, and some noticed effects, although some challenges with regard to the implementation and design remain.

Understanding the problems and the needs of the end-users for whom the product is developed is a core principle of UCD (62) and the main goal of the analysis phase of this project. Subsequently addressing the defined problem in a way that is meaningful to the user is a basic precondition for the usability of an intervention (64). Therefore, service users were consulted in various ways at all stages of the project. We found that service users with complex mental health problems were able to meaningfully participate and contribute to the understanding of the problem as well as to thinking about the form and content of the intervention. Even people who were less capable to express their needs and wishes regarding the content or design of the intervention were still able to indicate factors that they felt were important to consider. Apart from the service users, we also included other potential users and stakeholders (e.g., family, significant others, mental health workers, researchers, and rehabilitation professionals) in the UCD process. This revealed additional insights into the design criteria of the intervention that we formulated at the end of the analysis phase. These design criteria were used as a basis for developing a first prototype of TiM during the brainstorm session and were considered helpful by the participants. In addition, participants perceived the involvement of creative professionals during this session as helpful in order to stimulate thinking “out of the box.” Building upon the insights from the brainstorm session, as well as the subsequent input of focus groups with peer support workers, relatives, and psychiatric nurses, the six initial design criteria were supplemented to a total of eight design criteria (see Focus Groups Facilitating/Hampering Factors for Recovery). The subsequent iterative process of prototyping, testing, evaluating, and redesigning, which is typical of UCD [e.g., (63)], was crucial in adapting and fine-tuning TiM to the needs of the service users as well as mental health workers and family members.

In the pilot study, we investigated four main questions concerning the usage, effects, evaluation, and the implementation of the newly developed intervention. We found that TiM was used, but not with the frequency (biweekly) we originally suggested. People used TiM in various ways, in groups or in pairs, and inside and outside the service user's place of residence. For service users, it turned out important to use TiM in the presence of someone who took the lead, but who was supportive, made appointments, and helped suggest/choose activities. Results additionally suggest that people may abstain from using TiM because they initially had trouble to understand how to use TiM. Some people described their first impression of TiM as complicated, although once using it, almost everyone, including the professionals, found TiM understandable and enjoyable. The introduction event to acquaint people with TiM seemed to lower the barrier for the usage of TiM and helped people understand TiM. The introduction even also stimulated role patterns that deviated from the traditional role patterns, underlining the role of context in which TiM is offered. Upon using TiM, TiM pairs varied with regard to the amount of personal information that they exchanged. This was the case for both service users as well as mental health professionals and may be a matter of personal preference. Effects that were noticed by some participants particularly referred to the quality of the relationship between service users and professionals. Most people experienced that they had gotten closer, and there was more equality than before. One TiM pair used TiM to get acquainted with one another, which worked really well for them. Furthermore, we observed that TiM activated some service users. They demonstrated an increased interest to perform some new activities more often and took more initiative according to the professionals. Questions regarding identity were difficult to answer for most service users; most people did not notice changes. One person indicated to reflect more on himself/herself, whereas another person experienced to learn something new about himself/herself.

The results of the pilot study suggest that TiM may be a useful tool for peer support workers, as it fits well with their role. However, professionals indicated to expect a beneficial effect if other colleagues would use TiM as well, as they could see the benefit for the service users. However, they also expected barriers in this regard, given the many tasks already expected from nurses and caretakers, as well as the belief that an intervention such as TiM was not considered part of their professional profile. The implementation process of TiM thus requires continued attention within mental health teams. It was suggested that coupling of TiM to specific situations (e.g., intake procedure, new case manager) might facilitate implementation. However, as suggested by the service users, using TiM will not be suitable for all service users and thus should remain optional and up to the user.

Both with respect to the UCD development process and the evaluation of TiM, several discussion points can be raised. During the development process, the goal was to create a highly usable intervention, given the importance of usability for the implementation process [e.g., (64, 76)] and given the availability of many effective, but hardly used treatments (74, 75). An important factor in this regard was flexibility, as more flexible interventions tend to be better implementable (77). The importance of flexibility of an intervention is confirmed by the results of the focus group meeting with peer support workers, relatives, and psychiatric nurses in the design phase. In all focus groups, the necessity to adapt to the uniqueness of the individual was underlined. Moreover, the importance of accounting for individual differences in the process of treatment is at the very basis of recovery-oriented practice (17). The final product of the UCD process, TiM, contains high levels of flexibility. Although people are recommended to use TiM biweekly, they can alternate the frequency depending on their own preferences; the pilot study confirms that people indeed differ in their preferences regarding frequency. In addition, TiM includes an element of choice both in the selection of the activity and the manner in which the activity is executed. The pilot study indeed demonstrated variability in the use of TiM (pairs vs. groups), and choices regarding exact nature and location of activities were variable.

The current study confirmed the importance of minimizing the complexity of the intervention, to ensure usability and thus implementability (64). The iterative UCD process revealed the initial complexity of TiM and allowed us to further fine-tune and adapt the intervention. Nevertheless, despite the finding that the final product was perceived understandable and intuitive to all except one user in the pilot, initially a number of people initially believed TiM to be too complex. Although internalized stigma may have (partially) caused this experience (85), it does confirm the importance of the factor of complexity, as well as the importance of prototyping, testing, and redesigning.

When an intervention is found effective, this may facilitate the implementation success of an intervention (64). Although the effectiveness of TiM cannot be evaluated with the current data, the expectation of effectiveness of TiM that some service users and mental health workers expressed stimulated them to use or recommend TiM. In addition, some users reported to experience beneficial effects of the intervention. Interestingly, while the aim of TiM was to stimulate identity development in order to establish personal recovery, the most noticeable effects concerned the improved relationship with the TiM partner and activation of the service users. This may be due to the timely process of rebuilding a multidimensional identity (86), for which the duration of the pilot study may have been too short. Alternatively, changes in the relationship between TiM partners may be more noticeable and concrete, relative to changes in identity. However, it is also possible that changes in the relationship represent a first effect of identity development. Indeed, literature suggests that intergroup identity, which is an important part of self-identity, is something that often arises from a social context (18, 19). The equality that some people experienced as they progressed with the intervention may, in due time, impact the intergroup identity of the services users. In addition, newly gained experiences and roles, as a result of the employed activities, may contribute to the process of rebuilding this intergroup identity and contribute to establishing a sense of purpose. This latter point is particularly relevant for the current target population, people with SMI with both cognitive and communication challenges (24). Difficulties with self-reflection have been described for people with schizophrenia in general (23, 87, 88), and these may be even more substantial in this subgroup of service users. It is possible that changes in identity were too subtle to notice with the current measurements.

## Strengths and Weaknesses

Because of the relatively small sample size as well as the fact that not all participants used TiM, we may not have reached data saturation and thus may have missed opinions about TiM. This is something that we need to consider upon the continued UCD process in fine tuning TiM. Furthermore, we run the risk of a selection bias, as people were approached at the introduction event. For some service users, participating in an event like this may have been out of their comfort zone. Additionally, participants volunteered to participate in the study. Possibly people who were unwilling to participate had different opinions regarding the intervention. Although we attempted to involve family members in the implementation process, this was not successful. This may be due to the fact that many service users have lost contact over time with their families, and where there still is contact, people may not always be open to getting involved in the (89). However, there was diversity in the group of included participants. In addition, we included both the perspectives of service users and mental health professionals, which increases diversity of listed opinions. Furthermore, analysis and discussion were done by two authors who represent different perspectives, to improve thorough description and interpretation of the data. A strength of the study design was the uncontrolled usage of TiM (which is typically highly controlled in randomized controlled trials), which gave us a more realistic idea about the usage and implementation of TiM in clinical practice. An important lesson is that, while the UCD process will have substantially improve usability (and thus implementability), we still encountered implementation difficulties. For example, despite involvement of mental health nurses in the UCD process, many mental health nurses indicated that they had no time to use TiM. However, as one professional noted, some mental health workers did not consider TiM part of their task description, lowering the acceptability of TiM for these stakeholders. This observation may also represent a more general difficulty among mental health workers to shift from a symptom-oriented toward recovery-oriented practice. For now, interventions with the focus upon identity development and sense of purpose may be best allocated to peer support workers, who are specifically trained in this recovery-oriented perspective, or occupational therapists, who are involved in supporting service users in establishing different roles in life. Finally, although we developed TiM with a focus upon nonverbal communication, the measurements in the pilot were largely verbal. Upon this point, further research into the development of nonverbal measurement instruments is needed, to be equipped for studying the topic of identity in people with cognitive and communicative impairments.

With respect to the design of TiM we believe that improvements could still be made, particularly regarding the amount of available activities, the nonverbal nature of TiM design, and options for adaptability and personalization. Importantly, these drawbacks may be partly associated with the physical nature of the current design. Exploring the options for digitalization of TiM may be worthwhile in the continued UCD process. A digital interface facilitates sharing experiences with family members or others, and it would increase accessibility for a larger group of people because physical materials are not required. Furthermore, future endeavors may include extending TiM to other target population, who may experience similar struggles with self-identity and sense of purpose (e.g., people with traumatic brain injury or other life-changing physical or mental conditions). The major advantage of a UCD process is that it is circular, which enables us to take the input from the development process and the evaluation study as described in this article into account and use it for the development of an improved (digital) version of TiM in the future. A final strength of the study is that we were able to demonstrate that the process of participating in the UCD process itself contributed to the process of recovery in some service users. People indicated that they rediscovered their ability to help others with their input, a new meaningful role. As one service user put it: “finally somebody asked me to use my brain again.” Although not all service users will be interested or are able to contribute to these kinds of processes, the value of their contribution often remains unrecognized by many, or their capabilities may be underestimated as a result of stigma. Overall, we can conclude that UCD process is a useful and usable method for the development of a new psychosocial intervention, as well as increases the knowledge regarding factors that are important in supporting personal recovery for people with complex mental health needs.

## Data Availability Statement

The datasets generated for this article are not readily available because Participants did not consent to sharing their data with third parties. Requests to access the datasets should be directed to l.van.der.meer@rug.nl.

## Ethics Statement

The studies involving human participants were reviewed and approved by Medisch Ethische Toetsingscommissie UMCG. The patients/participants provided their written informed consent to participate in this study.

## Author Contributions

LvM was initiator and principle investigator of the study. TJ conducted the study in cooperation with HW, LvM, and CW. CW, JvW, and GP contributed to the project with additional scientific and clinical input to the UCD process. EvS conducted the qualitative pilot and data analysis in cooperation with LvM and TJ. LvM and EvS drafted a first version of the manuscript. All authors contributed to the article and approved the submitted version.

## Conflict of Interest

The authors declare that the research was conducted in the absence of any commercial or financial relationships that could be construed as a potential conflict of interest.

## References

[1] Killaspy H. The ongoing need for local services for people with complex mental health problems. Psychiatr Bull. (2014) 38:257–9. 10.1192/pb.bp.114.048470 PMC4248159 25505623

[2] Trieman N Leff J. Long-term outcome of long-stay psychiatric in-patients considered unsuitable to live in the community. TAPS Project 44. Br J Psychiatry. (2002) 181:428–32. 10.1192/bjp.181.5.428 12411270

[3] Wiersma D Nienhuis FJ Slooff CJ Giel R. Natural course of schizophrenic disorders: a 15-year followup of a Dutch incidence cohort. Schizophr Bull. (1998) 24:75–85. 10.1093/oxfordjournals.schbul.a033315 9502547

[4] Meltzer HY. Treatment-resistant schizophrenia—The role of clozapine. Curr Med Res Opin. (1997) 14:1–20. 10.1185/03007999709113338 9524789

[5] Gold JM Robinson B Leonard CJ Hahn B Chen S McMahon RP. Selective attention, working memory, and executive function as potential independent sources of cognitive dysfunction in schizophrenia. Schizophr Bull. (2018) 44:1227–34. 10.1093/schbul/sbx155 29140504 PMC6192492

[6] McEvoy JP Meyer JM Goff DC Nasrallah HA Davis SM Sullivan L. Prevalence of the metabolic syndrome in patients with schizophrenia: baseline results from the Clinical Antipsychotic Trials of Intervention Effectiveness (CATIE) schizophrenia trial and comparison with national estimates from NHANES III. Schizophr Res. (2005) 80:19–32. 10.1016/j.schres.2005.07.014 16137860

[7] Wiersma D Wanderling J Dragomirecka E Ganev K Harrison G Heiden W. Social disability in schizophrenia: its development and prediction over 15 years in incidence cohorts in six European centres. Psychol Med. (2000) 30:1155–67. 10.1017/S0033291799002627 12027051

[8] van Os J Kapur S. Schizophrenia. Lancet. (2009) 374:635–45. 10.1016/S0140-6736(09)60995-8 19700006

[9] Muthert JK. Ruimte Voor Verlies: Geestelijke Verzorging in de Psychiatrie: Vol. tweede serie. Tilburg: KSGV (2012).

[10] Anthony WA. Recovery from mental illness: The guiding vision of the mental health service system in the 1990s. Psychosoc Rehabil J. (1993) 16:11. 10.1037/h0095655

[11] van Weeghel J van Zelst C Boertien D Hasson-Ohayon I. Conceptualizations, assessments, and implications of personal recovery in mental illness: a scoping review of systematic reviews and meta-analyses. Psychiatr Rehabil J. (2019) 42:169–81. 10.1037/prj0000356 30843721

[12] Deegan PE. Recovery as a journey of the heart. Psychiatr Rehabil J. (1996) 19:91–7. 10.1037/h0101301

[13] Mancini MA Hardiman ER Lawson HA. Making sense of it all: Consumer providers' theories about factors facilitating and impeding recovery from psychiatric disabilities. Psychiatr Rehabil J. (2005) 29:48–55. 10.2975/29.2005.48.55 16075697

[14] Roe D Mashiach-Eizenberg M Lysaker PH. The relation between objective and subjective domains of recovery among persons with schizophrenia-related disorders. Schizophr Res. (2011) 131:133–8. 10.1016/j.schres.2011.05.023 21669512

[15] Yanos PT Roe D Lysaker PH. The Impact of illness identity on recovery from severe mental illness. Am J Psychiatr Rehabil. (2010) 13:73–93. 10.1080/15487761003756860 20802840 PMC2927828

[16] Bird V Leamy M Tew J Le Boutillier C Williams J Slade M. Fit for purpose? Validation of a conceptual framework for personal recovery with current mental health consumers. Aust N Zealand J Psychiatry. (2014) 48:644–53. 10.1177/0004867413520046 24413806

[17] Leamy M Bird V Le Boutillier C Williams J Slade M. Conceptual framework for personal recovery in mental health: systematic review and narrative synthesis. Br J Psychiatry. (2011) 199:445–52. 10.1192/bjp.bp.110.083733 22130746

[18] Tajfel H Turner JC. An integrative theory of intergroup conflict. In: Austin WG, Worchel S, editors. The Social Psychology of Intergroup Relations. Brooks/Cole (1979). p. 33–37.

[19] Turner JC Hogg MA Oakes PJ Reicher SD Wetherell MS. *Rediscovering the Social* GROUP: *A Self-Categorization Theory*. Oxford; New York, NY: Basil Blackwell (1987).

[20] Fukuyama F. Identity: The Demand for Dignity and the Politics of Resentment. 1st ed. New York, NY: Farrar, Straus and Giroux (2018).

[21] Hermans HJ Kempen HJ Van Loon RJ. The dialogical self: beyond individualism and rationalism. Am Psychol. (1992) 47:23–33. 10.1037/0003-066X.47.1.23

[22] Conneely M McNamee P Gupta V Richardson J Priebe S Jones JM. Understanding identity changes in psychosis: a systematic review and narrative synthesis. Schizophr Bull. (2020) sbaa124. 10.1093/schbul/sbaa124 32989443 PMC7965068

[23] van der Meer L Stiekema APM van Tol M-J Nolen WA David AS Aleman A. Insight in schizophrenia: Involvement of self-reflection networks? Schizophr Bull. (2013) 39:1288–95. 10.1093/schbul/sbs122 23104865 PMC3796073

[24] Fett A-KJ Viechtbauer W Dominguez M-G Penn DL van Os J Krabbendam L. The relationship between neurocognition and social cognition with functional outcomes in schizophrenia: a meta-analysis. Neurosci Biobehav Rev. (2011) 35:573–88. 10.1016/j.neubiorev.2010.07.001 20620163

[25] Deegan PE. Recovering our sense of value after being labeled: mentally ill. J Psychosoc Nurs Mental Health Serv. (1993) 31:7–9. 10.3928/0279-3695-19930401-06 8487230

[26] van Mierlo T van der Meer L Voskes Y Berkvens B Stavenuiter B van Weeghel J. Werkboek Active Recovery Triad. Utrecht: De Tijdstroom (2016). 10.3389/fpsyt.2020.592228 PMC7674651 33250796

[27] Castelein S. Overstag en Vooruit! Herstelbevordering bij Ernstige Psychische Aandoeningen. (2018). Available online at: https://www.youtube.com/watch?v=vfoSSBclJIQ (accessed February 28, 2021).

[28] De Heer-Wunderink C Visser E Sytema S Wiersma D. Social inclusion of people with severe mental illness living in community housing programs. Psychiatr Serv. (2012) 63:1102–7. 10.1176/appi.ps.201100538 22948812

[29] Jackson L Tudway JA Giles D Smith J. An exploration of the social identity of mental health inpatient service users. J Psychiatr Mental Health Nurs. (2009) 16:167–76. 10.1111/j.1365-2850.2008.01361.x 19281548

[30] Yanos PT DeLuca JS Roe D Lysaker PH. The impact of illness identity on recovery from severe mental illness: a review of the evidence. Psychiatry Res. (2020) 288:112950. 10.1016/j.psychres.2020.112950 32361335

[31] Leonhardt BL Huling K Hamm JA Roe D Hasson-Ohayon I McLeod HJ. Recovery and serious mental illness: a review of current clinical and research paradigms and future directions. Exp Rev Neurother. (2017) 17:1117–30. 10.1080/14737175.2017.1378099 28885065

[32] Lysaker PH Davis LW Eckert GJ Strasburger AM Hunter NL Buck KD. Changes in narrative structure and content in schizophrenia in long term individual psychotherapy: a single case study. Clin Psychol Psychother. (2005) 12:406–16. 10.1002/cpp.457

[33] Lysaker PH Davis LW Jones AM Strasburger AM Beattie NL. Relationship and technique in the long-term integrative psychotherapy of schizophrenia: a single case study. Counsel Psychother Res. (2007) 7:79–85. 10.1080/14733140701345869

[34] van der Meer L Wunderink C. Contemporary approaches in mental health rehabilitation. Epidemiol Psychiatr Sci. (2019) 28:9–14. 10.1017/S2045796018000343 30043719 PMC6998946

[35] Farkas M Jansen MA Penk WE. Psychosocial rehabilitation: Approach of choice for those with serious mental illnesses. J Rehabil Res Dev. (2007) 44:vii–xxi. 10.1682/JRRD.2007.09.0143 18075935

[36] Sanches SA Swildens WE Schaefer B Moerbeek M Feenstra TL van Asselt ADI. Effectiveness of the Boston university approach to psychiatric rehabilitation in improving social participation in people with severe mental illnesses: a randomized controlled trial. Front Psychiatry. (2020) 11:571640. 10.3389/fpsyt.2020.571640 33173519 PMC7538503

[37] Swildens W van Busschbach JT Michon H Kroon H Koeter MWJ Wiersma D. Effectively working on rehabilitation goals: 24-Month outcome of a randomized controlled trial of the Boston Psychiatric Rehabilitation Approach. Can J Psychiatry. (2011) 56:751–60. 10.1177/070674371105601207 22152644

[38] Rapp CA Goscha RJ. The Strengths Model: A Recovery-Oriented Approach to Mental Health Services. Oxford; New York, NY: Oxford University Press (2011).

[39] Allott K van-der-EL K Bryce S Parrish EM McGurk SR Hetrick S. Compensatory interventions for cognitive impairments in psychosis: a systematic review and meta-analysis. Schizophr Bull. (2020) 46:869–83. 10.1093/schbul/sbz134 32052837 PMC7345816

[40] Bowie CR Bell MD Fiszdon JM Johannesen JK Lindenmayer J-P McGurk SR. Cognitive remediation for schizophrenia: an expert working group white paper on core techniques. Schizophr Res. (2020) 215:49–53. 10.1016/j.schres.2019.10.047 31699627

[41] Deenik J Kruisdijk F Tenback D Braakman-Jansen A Taal E Hopman-Rock M. Physical activity and quality of life in long-term hospitalized patients with severe mental illness: a cross-sectional study. BMC Psychiatry. (2017) 17:298. 10.1186/s12888-017-1466-0 28821287 PMC5562976

[42] Looijmans A Stiekema APM Bruggeman R Meer L van der Stolk RP Schoevers RA. Changing the obesogenic environment to improve cardiometabolic health in residential patients with a severe mental illness: cluster randomised controlled trial. Br J Psychiatry. (2017) 211:296–303. 10.1192/bjp.bp.117.199315 28982656

[43] Burns T Catty J Becker T Drake RE Fioritti A Knapp M. The effectiveness of supported employment for people with severe mental illness: a randomised controlled trial. Lancet. (2007) 370:1146–52. 10.1016/S0140-6736(07)61516-5 17905167

[44] Ringeisen H Langer Ellison M Ryder-Burge A Biebel K Alikhan S Jones E. Supported education for individuals with psychiatric disabilities: state of the practice and policy implications. Psychiatr Rehabil J. (2017) 40:197–206. 10.1037/prj0000233 28182470

[45] Fukui S Starnino VR Susana M Davidson LJ Cook K Rapp CA. Effect of wellness recovery action plan (WRAP) participation on psychiatric symptoms, sense of hope, and recovery. Psychiatr Rehabil J. (2011) 34:214–22. 10.2975/34.3.2011.214.222 21208860

[46] van Gestel-Timmermans H Brouwers EPM van Assen MALM van Nieuwenhuizen C. Effects of a peer-run course on recovery from serious mental illness: a randomized controlled trial. Psychiatr Serv. (2012) 63:54–60. 10.1176/appi.ps.201000450 22227760

[47] Boevink W Kroon H van Vugt M Delespaul P van Os J. A user-developed, user run recovery programme for people with severe mental illness: a randomised control trial. Psychosis. (2016) 8:287–300. 10.1080/17522439.2016.1172335

[48] Nijman SA Veling W Greaves-Lord K Vos M Zandee CER Aan Het Rot M. Dynamic Interactive Social Cognition Training in Virtual Reality (DiSCoVR) for people with a psychotic disorder: Single-Group feasibility and acceptability study. JMIR Mental Health. (2020) 7:e17808. 10.2196/17808 32763880 PMC7442939

[49] Rus-Calafell M Garety P Sason E Craig TJK Valmaggia LR. Virtual reality in the assessment and treatment of psychosis: a systematic review of its utility, acceptability and effectiveness. Psychol Med. (2018) 48:362–91. 10.1017/S0033291717001945 28735593

[50] Yanos PT Roe D Lysaker PH. Narrative enhancement and cognitive therapy: a new group-based treatment for internalized stigma among persons with severe mental illness. Int J Group Psychother. (2011) 61:576–95. 10.1521/ijgp.2011.61.4.576 21985260 PMC3191919

[51] Roe D Hasson-Ohayon I Mashiach-Eizenberg M Derhy O Lysaker PH Yanos PT. Narrative enhancement and cognitive therapy (NECT) effectiveness: a quasi-experimental study. J Clin Psychol. (2014) 70:303–12. 10.1002/jclp.22050 24114797 PMC3954406

[52] Roe D Hasson-Ohayon I Mashiach-Eizenberg M Yamin A Lysaker PH. Different roads lead to Rome: exploring patterns of change among Narrative Enhancement and Cognitive Therapy (NECT) participants. Isr J Psychiatry Relat Sci. (2017) 54:62–70. 28857760

[53] Yanos PT Lysaker PH Silverstein SM Vayshenker B Gonzales L West ML. A randomized-controlled trial of treatment for self-stigma among persons diagnosed with schizophrenia-spectrum disorders. Soc Psychiatr Psychiatr Epidemiol. (2019) 54:1363–78. 10.1007/s00127-019-01702-0 30937510 PMC6773531

[54] Yanos PT Roe D West ML Smith SM Lysaker PH. Group-based treatment for internalized stigma among persons with severe mental illness: findings from a randomized controlled trial. Psychol Serv. (2012) 9:248–58. 10.1037/a0028048 22545821 PMC3413784

[55] van Donkersgoed RJ de Jong S Van der Gaag M Aleman A Lysaker PH Wunderink L. A manual-based individual therapy to improve metacognition in schizophrenia: protocol of a multi-center RCT. BMC Psychiatry. (2014) 14:27. 10.1186/1471-244X-14-27 24490942 PMC3922090

[56] de Jong S Donkersgoed RJM van Timmerman ME Rot M aan het Wunderink L Arends J. Metacognitive Reflection and Insight Therapy (MERIT) for patients with schizophrenia. Psychol Med. (2019) 49:303–13. 10.1017/S0033291718000855 29692285

[57] de Jong S Hasson-Ohayon I Donkersgoed R van Aleman A Pijnenborg GHM. A qualitative evaluation of the effects of Metacognitive Reflection and Insight Therapy: ‘Living more consciously.’ Psychol Psychother Theory Res Pract. (2020) 93:223–40. 10.1111/papt.12212 30548375

[58] Meadows G Brophy L Shawyer F Enticott JC Fossey E Thornton CD. REFOCUS-PULSAR recovery-oriented practice training in specialist mental health care: A stepped-wedge cluster randomised controlled trial. Lancet Psychiatry. (2019) 6:103–14. 10.1016/S2215-0366(18)30429-2 30635177

[59] Davidson M. Cognitive impairment as a diagnostic criterion and treatment target in schizophrenia. World Psychiatry. (2019) 18:171–2. 10.1002/wps.20651 31059612 PMC6502436

[60] Werremeyer A Skoy E Burns W Bach-Gorman A. Photovoice as an intervention for college students living with mental illness: a pilot study. Mental Health Clin. (2020) 10:237–43. 10.9740/mhc.2020.07.237 32685335 PMC7337996

[61] Sitvast J. Narrative and meaning of life: how mental health nurses can respond. Psychol Psychother Res Study. (2018) 1:1–7. 10.31031/PPRS.2018.01.000503

[62] Cooper A Reimann R Cronin D Noessel C. About Face: The Essentials of Interaction Design. John Wiley & Sons, Incorporated (2014).

[63] Altman M Huang TTK Breland JY. Design thinking in health care. Prev Chronic Dis. (2018) 15. 10.5888/pcd15.180128 PMC6178900 30264690

[64] Lyon AR Koerner K. User-Centered design for psychosocial intervention development and implementation. Clin Psychol Sci Pract. (2016) 23:180–200. 10.1111/cpsp.12154 29456295 PMC5812700

[65] Roberts JP Fisher TR Trowbridge MJ Bent C. A design thinking framework for healthcare management and innovation. Healthcare. (2016) 4:11–4. 10.1016/j.hjdsi.2015.12.002 27001093

[66] Graham AK Wildes JE Reddy M Munson SA Taylor CB Mohr DC. User-centered design for technology-enabled services for eating disorders. Int J Eating Disord. (2019) 52:1095–107. 10.1002/eat.23130 31313370 PMC7265747

[67] Hardy A Wojdecka A West J Matthews E Golby C Ward T. How inclusive, user-centered design research can Improve psychological therapies for psychosis: development of SlowMo. JMIR Mental Health. (2018) 5:e11222. 10.2196/11222 30518514 PMC6300708

[68] Pelletier J-F Rowe M François N Bordeleau J Lupien S. No personalization without participation: on the active contribution of psychiatric patients to the development of a mobile application for mental health. BMC Med Inform Decis Making. (2013) 13:78. 10.1186/1472-6947-13-78 23890085 PMC3729542

[69] Lyon AR Bruns EJ. User-Centered redesign of evidence-based psychosocial interventions to enhance implementation—Hospitable soil or better seeds? JAMA Psychiatry. (2019) 76:3–4. 10.1001/jamapsychiatry.2018.3060 30427985

[70] Chorpita BF Daleiden EL. Structuring the collaboration of science and service in pursuit of a shared vision. J Clin Child Adolesc Psychol. (2014) 43:323–38. 10.1080/15374416.2013.828297 23981145

[71] Chambers DA Glasgow RE Stange KC. The dynamic sustainability framework: addressing the paradox of sustainment amid ongoing change. Implement Sci. (2013) 8:1–11. 10.1186/1748-5908-8-117 24088228 PMC3852739

[72] Drake RE Goldman HH Leff HS Lehman AF Dixon L Mueser KT. Implementing evidence-based practices in routine mental health service settings. Psychiatr Serv. (2001) 52:179–82. 10.1176/appi.ps.52.2.179 11157115

[73] Moir T. Why is implementation science important for intervention design and evaluation within educational settings? Front Educ. (2018) 3:61. 10.3389/feduc.2018.00061

[74] Lehman AF Steinwachs DM. Translating research into practice: the schizophrenia Patient Outcomes Research Team (PORT) treatment recommendations. Schizophr Bull. (1998) 24:1–10. 10.1093/oxfordjournals.schbul.a033302 9502542

[75] van Duin D Franx G van Wijngaarden B van Der Gaag M van Weeghel J Slooff C. Bridging the science-to-service gap in schizophrenia care in the Netherlands: the Schizophrenia Quality Improvement Collaborative. Int J Qual Health Care. (2013) 25:626–32. 10.1093/intqhc/mzt072 24179181

[76] Lyon AR Wasse JK Ludwig K Zachry M Bruns EJ Unützer J. The Contextualized Technology Adaptation Process (CTAP): optimizing health information technology to improve mental health systems. Administr Policy Mental Health Mental Health Serv Res. (2016) 43:394–409. 10.1007/s10488-015-0637-x 25677251 PMC4536193

[77] Cohen DJ Crabtree BF Etz RS Balasubramanian BA Donahue KE Leviton LC. Fidelity versus flexibility: translating evidence-based research into practice. Am J Prev Med. (2008) 35(5, Suppl.):S381–9. 10.1016/j.amepre.2008.08.005 18929985

[78] Dagnan D. Psychosocial interventions for people with intellectual disabilities and mental ill-health. Curr Opin Psychiatry. (2007) 20:456–60. 10.1097/YCO.0b013e3282ab9963 17762587

[79] Hatton C. Psychosocial interventions for adults with intellectual disabilities and mental health problems: a review. J Mental Health. (2002) 11:357–74. 10.1080/09638230020023732

[80] Proctor E Silmere H Raghavan R Hovmand P Aarons G Bunger A. Outcomes for implementation research: conceptual distinctions, measurement challenges, and research agenda. Administr Policy Mental Health. (2011) 38:65–76. 10.1007/s10488-010-0319-7 20957426 PMC3068522

[81] Farkas M Anthony WA. Psychiatric rehabilitation interventions: a review. Int Rev Psychiatry. (2010) 22:114–29. 10.3109/09540261003730372 20504052

[82] Cook JA Jonikas JA Hamilton MM Goldrick V Steigman PJ Grey DD. Impact of wellness recovery action planning on service utilization and need in a randomized controlled trial. Psychiatr Rehabil J. (2013) 36:250–7. 10.1037/prj0000028 24320833

[83] Copeland ME. Wellness Recovery Action Plan. A system for monitoring, reducing and eliminating uncomfortable or dangerous physical symptoms and emotional feelings. Occup Ther Mental Health. (2002) 17:127–50. 10.1300/J004v17n03_09

[84] Friese S. Qualitative Data Analysis With ATLAS.ti. 3rd ed. London: Sage Publications Ltd. (2019).

[85] Corrigan PW Larson JE Rüsch N. Self-stigma and the “why try” effect: impact on life goals and evidence-based practices. World Psychiatry. (2009) 8:75–81. 10.1002/j.2051-5545.2009.tb00218.x 19516923 PMC2694098

[86] Lysaker PH Glynn SM Wilkniss SM Silverstein SM. Psychotherapy and recovery from schizophrenia: a review of potential applications and need for future study. Psychol Serv. (2010) 7:75–91. 10.1037/a0019115 20526422 PMC2880514

[87] Bonfils KA Luther L George S Buck KD Lysaker PH. The role of metacognitive self-reflectivity in emotional awareness and subjective indices of recovery in schizophrenia. J Nervous Mental Dis. (2016) 204:903–8. 10.1097/NMD.0000000000000599 27668353 PMC5125882

[88] Bonfils KA Minor KS Leonhardt BL Lysaker PH. Metacognitive self-reflectivity moderates the relationship between distress tolerance and empathy in schizophrenia. Psychiatry Res. (2018) 265:1–6. 10.1016/j.psychres.2018.04.042 29679792 PMC6309172

[89] Zomer LJC Voskes Y van Weeghel J Widdershoven GAM van Mierlo TFMM Berkvens BS. The active recovery triad model: a new approach in Dutch long-term mental health care. Front Psychiatry. (2020) 11:592228. 10.3389/fpsyt.2020.592228 33250796 PMC7674651

