# Supplementary material for: Targeting Personal Recovery of People With Complex Mental Health Needs: The Development of a Psychosocial Intervention Through User-Centered Design
Source: Front Psychiatry. 2021 Apr 8;12:635514. doi: 10.3389/fpsyt.2021.635514 (PMC8060492; doi:10.3389/fpsyt.2021.635514)
Supplement: Supplementary file 1 [file Data_Sheet_1.PDF]

Supplementary Material S1

Example of the travelogue.

Instructions

REISINSTRUCTIES

1. Kies iemand met wie je op reis wilt
2. Draai aan het rad en kies één van de drie reisbestemmingen Je mag maximaal 2 x draaien
3. Ervaar de reisbestemming samen met jouw medereiziger
4. Leg elke reisbestemming vast in een reisverslag met een foto een plaatje/tekening of een souvenir

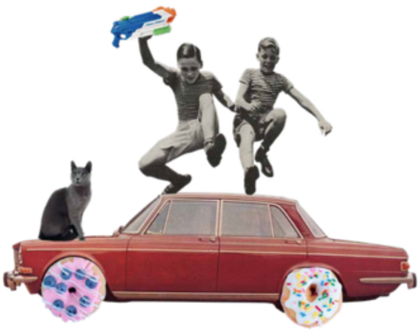

SPELREGELS

- Je mag verschillende mensen meenemen op jouw ontdekkingsreis
- Je mag maximaal één keer opnieuw draaien
- De bestemming is voor beide reizigers. Dit betekent dat één reisbestemming bestaat uit twee ervaringen
- In 5 reisbestemmingen moet minimaal één oranje, één groene en één gele bestemming bevatten
- Bezoek minimaal 6 unieke reisbestemmingen en vul dan met één van de medereizigers het 'dit ben ik' blad van jouw reisverslag in. Voeg zo veel reisbestemmingen toe als je wilt. Wij geloven dat meer reisbestemmingen altijd beter zijn
- Jouw reis is nooit af. Je kunt op elk moment nieuwe reisbestemmingen aan jouw reis toevoegen
- Op www.deontdekkingsreisvanmijnleven.nl staat meer informatie en video's die je helpen de reis nog beter te begrijpen

Travel instructions:
1. Pick a parter with whom you would like to take a journey
2. Spin the wheel and pick a travel destination. You can spin twice.
3. Experience the your journey together with your partner
4. Log your journey in this travelogue using a photo/picture/drawing or souvenir

Playing rules:
- You can take different people on your journeys
- You can spin one additional time
- the travel destination counts for both travelers. So one spin will entail two journeys
- In five travel destinations you need to inlcude at least one orange, one green and one yellow destination
- Visit at least 6 unique destinations and add this to your travelogue
- Your journey never ends. You can always add new destinations
- See www.deontdekkingsreisvanmijnleven.nl for information that will help you understand your journey.

Example of a page logging one activity/journey

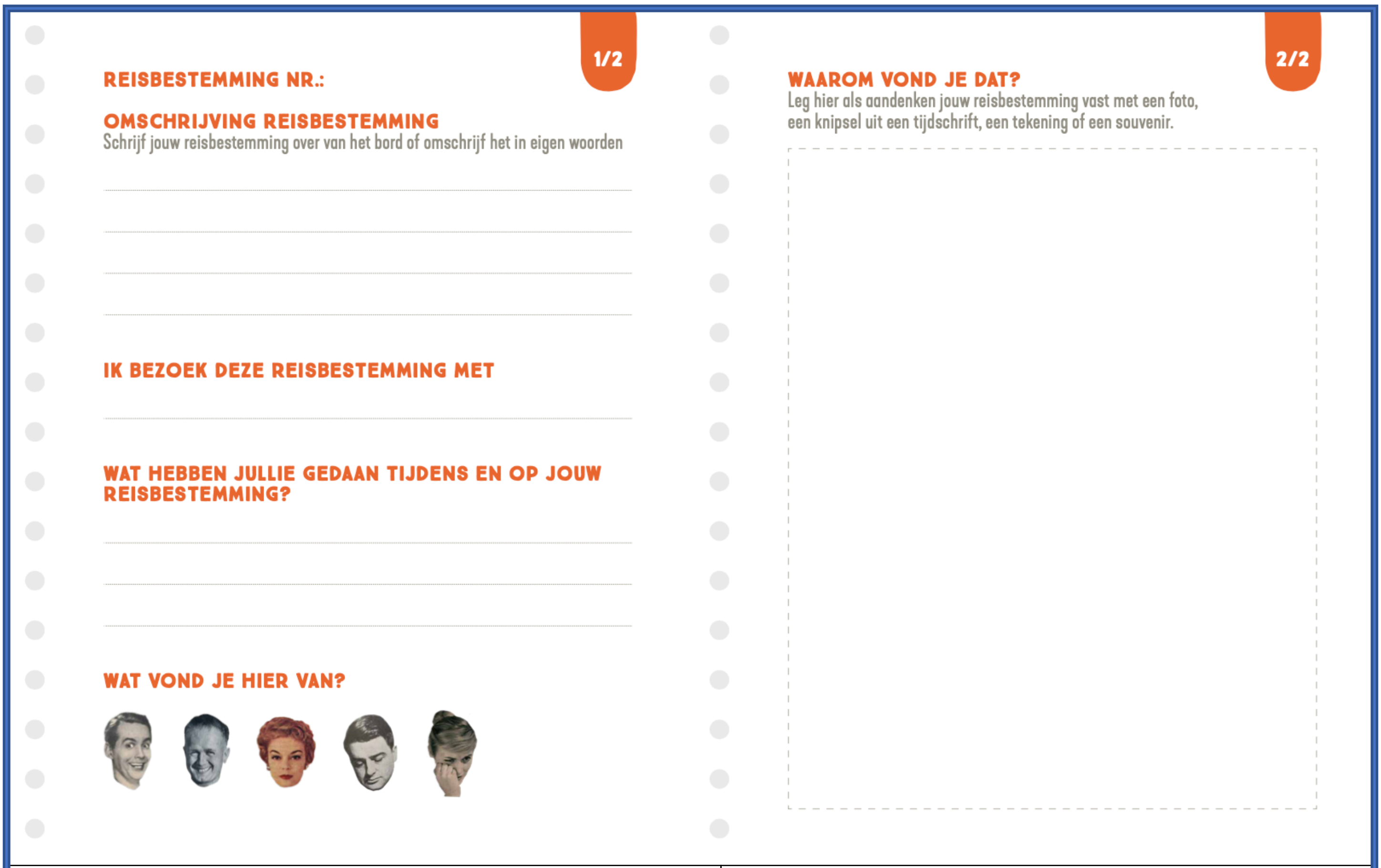

1/2

REISBESTEMMING NR.:

OMSCHRIJVING REISBESTEMMING
Schrijf jouw reisbestemming over van het bord of omschrijf het in eigen woorden

IK BEZOEK DEZE REISBESTEMMING MET

WAT HEBBEN JULLIE GEDAAN TIJDENS EN OP JOUW REISBESTEMMING?

WAT VOND JE HIER VAN?

2/2

WAAROM VOND JE DAT?
Leg hier als aandenken jouw reisbestemming vast met een foto, een knipsel uit een tijdschrift, een tekening of een souvenir.

**Destination Nr:**<br><br>**Description travel destination**<br>Copy your travel destination from the wheel or describe in your own words<br><br>________________<br><br>I will visit this travel destination with<br><br>________________<br><br>What did you do during and at your travel destination?<br><br>________________<br><br>How did you like this?	**Why did you experience this as such?**<br>Provide a souvenir of your travel destination here with a photo, picture (from a magazine/paper), drawing or other souvenir.

Final list of activities included in the picker wheel

Subcategory	Level 1	Level 2	Level 3
**Leisure time**	Show one another your favorite television show of the past	Do something together which you think the other person will enjoy	Visit a place together that reminds you of your favorite holiday destination
**Learning**	Teach the other person something you are (used to be) very good at	Set a first step in learning something that you would like to learn	Do something together that you have not done before
**Music**	Let each other listen to the music that was your favorite in the past	Find a musical instrument to play	Let the other person experience the music that you like best
**Significant other**	Show each other a photograph of a person who is important in your life	Write a postcard to someone you have not seen in a long time and with whom you would like to reestablish contact	Invite a new person with whom you would like to conduct an activity
**Characteristics**	Write down or dray three good characteristics that you see in the other person	Find an object that fits with the person you are and explain why to the other person	Find three objects that you think fit the other person and explain why
**Health**	Find a photo/picture that depicts the meaning of 'health' according to you	Have the other person experience what helps you to feel better when you are feeling off	Go and practice the sport that was your favorite in the past
**Family**	Make a drawing of you and your family that you can demonstrate to the other person	Write of draw a message that you would like to send to a family member	Have the other person experience an activity that you liked doing in the past with a family member
**Smell or taste**	Find (or create) a scent of which you have good memories and share this with the other person	Go out to smell different scents and explain to the other person how you feel about this scent	Have the other person smell or taste which food/drink you liked to eat/drink in the past
**Profession**	Find three professions in a magazine and both pick which profession you like best	Find someone with a profession that appeals to you and talk to him/her about his profession	What profession would you like to pursue these days? Take a first step to get closer to this goal
**House of the past**	Make a drawing of the house where you used to live or of another nice place from the last to show this to the other person	Show each other a picture of the city/village/place where you would like to live	Visit the house where you used to live or another place that is important to you
**Prejudice**	Find a vido on 'stigma' on the internet and watch this together	Show in a drawing what other people see when they look at you. Is this correct according to you and the other person?	Go out and as three new people to name a good feature of you
**Happy place**	Show each other the place where you feel most comfortable	Go out and show the other person a house that you really like	Visit a place from the past that is (used to be) important to you
**Happy things**	Go out for a walk and take a picture of something you find pretty	Find a plant/tree/flower you find pretty	Visit a place that you might like, but where you have never been
**Talent**	Find three different pictures that depict one of your talents	What used to be a talent of yours? Have the other person experience this	Go out and do something together that requires you to use a talent of yours
**Memories**	Show the other person pictures (of the past) that you hold happy memories from	When did you feel most like 'yourself'? Have the other person experience this	Visit the place together where you used to go to school.
